# Medical Students in France and the United States

**Published:** 1847-01

**Authors:** 


*Medical Students in France and the United States.*—In the United States, with a population of 20,000,000 of people, there are about thirty medical schools, and an average annual number of 4,500 students, 1,300 of whom are yearly graduated. In France, with a population of 35,000,000, there are but three medical schools, which graduate only about 700 annually.—*Dr. Stillé's Address.*

